# Fermentable Oligo-, Di-, and Mono-Saccharides and Polyols (FODMAPs) Consumption and Irritable Bowel Syndrome in the French NutriNet-Santé Cohort

**DOI:** 10.3390/nu13124513

**Published:** 2021-12-17

**Authors:** Elodie Schneider, Jean-Marc Sabaté, Michel Bouchoucha, Serge Hercberg, Mathilde Touvier, Robert Benamouzig, Chantal Julia, Camille Buscail

**Affiliations:** 1 Nutritional Epidemiology Research Team (EREN), Sorbonne Paris Cité Epidemiology and Statistics Research Center (CRESS), Inserm U1153, Inrae U1125, Cnam, Université Sorbonne Paris Nord University, 93017 Bobigny, France; s.hercberg@eren.smbh.univ-paris13.fr (S.H.); m.touvier@eren.smbh.univ-paris13.fr (M.T.); c.julia@eren.smbh.univ-paris13.fr (C.J.); cdebrauer@institutcancer.fr (C.B.); 2 Service de Gastroentérologie, Hôpital Avicenne, APHP, 93017 Bobigny, France; jean-marc.sabate@aphp.fr (J.-M.S.); michel.bouchoucha@aphp.fr (M.B.); robert.benamouzig@aphp.fr (R.B.); 3 INSERM U-987, Hôpital Ambroise Paré (APHP), 92104 Boulogne-Billancourt, France; 4 Département de Santé Publique, Hôpital Avicenne (APHP), 93017 Bobigny, France

**Keywords:** IBS, FODMAP intake, cross-sectional study, Rome IV criteria, Nutrinet-Santé cohort study

## Abstract

(1) Background: Specific foods, and more particularly, fermentable oligo-, di-, and mono-saccharides and polyols (FODMAPs) are often considered as triggers of digestive symptoms in Irritable Bowel Syndrome (IBS). Our aim was to study FODMAP consumption in controls and IBS participants in a large French population-based cohort; (2) Methods: Participants from the NutriNet-Santé cohort study completed the Rome IV and IBS-SSS questionnaire in a cross sectional study. Among them, 27,949 eligible participants had previously completed three 24-h recalls as well as anthropometrics, socio-demographical and lifestyle data. Total FODMAP intake (in g/day) was computed using a specific composition table. The association between FODMAPs and IBS was estimated through multivariable logistic regression models; (3) Results: Included participants were mainly women (75.4%) and the mean age was 43.4 ± 14.1 years. FODMAPs accounted for a mean daily intake of 19.4 ± 9.5 g/day. Overall 1295 participants (4.6%) were identified with an IBS. After adjusting for confounding factors, IBS participants had lower intakes in FODMAPs than non-IBS ones (aOR: 0.88, 95% CI: 0.82–0.95, *p*-value: 0.001). IBS severity was associated with more frequent low FODMAP intakes (<9 g/day); (4) Conclusions: Participants tended to consume 19 g of FODMAPs per day, but slightly less for IBS participants than for controls. In IBS participants, higher severity was associated with lower intakes.

## 1. Introduction

Fermentable oligo-, di-, and mono-saccharides and polyols (FODMAPs) are short chain carbohydrates that are poorly absorbed in the gastrointestinal tract [1]. They include fructose in excess of glucose, lactose, fructans, galacto-oligo-saccharides (GOS) and polyols. They are ubiquitous in our diet, in particular through fruits, vegetables, legumes, dairy products, cereal products, oilseeds, seasonings, sugary products, alcoholic beverages, sugar-free products or sweeteners, and many processed items [2,3,4,5].

Among functional gastrointestinal disorders (FGIDs), irritable bowel syndrome (IBS) is one of the most prevalent and has therefore been of interest in recent years, affecting about 6–11% of the general population depending on the diagnostic criteria [6,7,8]. On this point, the Rome foundation recently updated the criteria used to diagnose IBS with the Rome IV criteria [9,10]. IBS, which is defined by chronic abdominal pain associated with a change in bowel habits, can be responsible for an impairment in quality of life of patients with severe disease and for an economic burden [11,12]. While IBS pathophysiology remains unclear, nutrient and non-nutrient components of foods, including FODMAPs, are often suspected to trigger IBS symptoms such as abdominal pain, bloating, excessive passage of gas and also diarrhea [13,14,15,16]. In recent years, several clinical trials have suggested that a low FODMAP diet (LFD) may be associated with a reduction in IBS symptoms [5,17,18,19,20,21].

Reviews on this association expressed a lack of large-scale data [22,23,24]. Moreover, comparisons between FODMAP consumption in IBS participants and control subjects are lacking; only Halmos et al. described similar levels of daily intakes in a study with 30 IBS patients and 8 healthy subjects [5].

The main aim of this work was to study FODMAP consumption in both IBS participants and in controls and to assess the relationships between FODMAP load, IBS symptoms and severity in a large national cohort (NutriNet-Santé).

## 2. Materials and Methods

### 2.1. Population

The NutriNet-Santé study is a web-based prospective observational cohort study that includes French adults from the general population (more than 160,000 recruited to date). Detailed description of the cohort is available elsewhere [25]. Briefly, it aims at studying the relations between nutrition and health, as well as determinants of dietary patterns and nutritional status [25]. Inclusions started in May 2009 and were still ongoing at the time of this study. At baseline, participants completed a set of five questionnaires about socio-demographic and lifestyle characteristics, anthropometry, health status, physical activity and diet. These questionnaires were repeated yearly. Furthermore, during follow-up, additional questionnaires on other specific topics dealing with determinants of food behaviors or health were regularly sent to participants.

### 2.2. Ethics

This study was conducted in accordance with the Declaration of Helsinki, and was approved by the Institutional Review Board of the French Institute for Health and Medical Research (IRB Inserm 0000388FWA00005831) and the “Commission Nationale de l’Informatique et des Libertés” (CNIL n 908450 and 909216). The study is registered at clinicaltrials.gov (accessed on 21 May 2019) as NCT03335644. All participants provided an electronic informed consent.

### 2.3. Data Collection

#### 2.3.1. Irritable Bowel Syndrome

An additional questionnaire was sent to the cohort in April 2018 to assess gastrointestinal disorders. History of digestive diseases and symptoms were collected using the Rome IV questionnaire to define IBS (with symptoms for at least 6 months) [26]. The questionnaire also included several questions about digestive medical history and symptoms. Thus, people were not eligible to participate in this study if they had had a history of rectal bleeding, melena, hematemesis, or significant unintentional weight loss (>5 kg) in the past 3 months, as well as a history of oesophagus, stomach or colorectal cancer, coeliac disease or ulcerative colitis in their family, as these symptoms were considered proxies for an organic underlying condition.

#### 2.3.2. Diet

Participants were requested to fill a set of three 24-h diet recall interviews at baseline and every 6 months (three non-consecutive days over a fortnight including two on weekdays and one during a weekend) [25]. Food record relied on a meal-based approach, asking nature and sizes of the portions of food eaten at each occasion (including meals and snacking periods). Portion sizes were assessed using validated photographs [27], and dietary intakes were based on the NutriNet-Santé food composition table [28]. This self-administered food record method has been validated in several studies in comparison to urinary biomarkers and interview by a dietitian [29,30,31]. In this work, the first full set of food records available was used to estimate dietary intakes for each individual.

A specific composition table was used to assess FODMAP consumption [32]. This table enables to compute total FODMAP intake (in weight, grams/day), as well as each of: fructose in excess of glucose (excess-fructose), lactose, fructans, GOS and polyols for each participant. For the analyses, total FODMAP intakes were divided into three categories (<9 g/day, 9–16 g/day, ≥16 g/day) according to previous works published on low and usual FODMAP diet [5,33,34]. Eligible participants for this study had completed at least one full set of three 24-h food records after the inclusion and prior to the Rome IV questionnaire. Under-reporters of energy intake, identified through the Black method, were excluded from this study [35].

#### 2.3.3. IBS Scoring System

The IBS Symptom Scoring System (IBS-SSS) was used to assess IBS severity in participants with IBS diagnosis based on the Rome IV questionnaire. Other participants were also asked to fill this scoring system if they were previously diagnosed with an IBS or if they suspected they suffered from IBS (based on self-report). The IBS-SSS is divided into 5 main items (abdominal pain severity and frequency, abdominal distension severity, bowel habit satisfaction and impact on quality of life), ranged 1–100 each. The maximal achievable score is 500, mild, moderate and severe cases are indicated by scores of 75 to 175, 175 to 300 and >300 respectively. This score has been validated and described in detail elsewhere [36].

#### 2.3.4. Covariates

Information on socio-demographic characteristics was collected at baseline: age, sex, marital status (single/living with someone), smoking status (current smoker/former smoker/non-smoker), monthly income and composition of the household enabling to calculate monthly income per consumption unit (c.u.) (unwilling to answer/<1110 € per c.u./1110–2330 € per c.u./>2330 € per c.u.) [37], educational level (no diploma or primary school/secondary school/undergraduate school/graduate school) and residence (rural area/urban area). Self-reported weight and height at baseline were used to calculate body mass index (BMI, in kg/m^2^) (<18.5/18.5–25/25–30/≥30) [38,39,40]. A specific questionnaire was administered at baseline to assess physical activity level using the International Physical Activity Questionnaire (IPAQ, Intense/Moderate/Low) [41]. Total energy intake was computed (in kcal/day).

### 2.4. Statistical Analyses

A description of socio-demographic, lifestyle and anthropometric characteristics, health status, physical activity, overall diet quality and FODMAP consumption (total and individually) was performed on the whole sample, and according to the IBS status (yes/no). Chi-square tests or Student’s *t*-tests were used according to the qualitative or quantitative status of the variable.

In order to adjust for the overall diet quality, a principal component analysis was performed using food groups from the study population. The first dietary pattern was positively correlated with fruits and vegetables, whole grains, natural oilseeds, vegetable fat, non-sugared beverages, legumes and broth. It was negatively correlated with milky desserts, meat and ham, sweetened beverages, cakes, cookies and pastries, bread and processed meat. Therefore, it was labelled a “healthy” dietary pattern as previously published [42,43] and this dimension was used as a proxy for the overall diet quality. The estimate of the first principal component was divided into 3 categories (poor quality/intermediate quality or healthy diet).

Multivariate logistic regression models were applied to assess Odds Ratio (OR) and adjusted OR (aOR) with their 95% confidence intervals (95% CI) of IBS across FODMAP consumption. Models were successively adjusted for age and sex (model 2) as well as energy, BMI, income per consumption unit, marital status, smoking status, overall diet quality, IPAQ, residence and educational level (model 3). All tests were two-sided and a *p*-value < 0.05 was considered statistically significant. Statistical analyses were carried out using SAS software (version 9.4; SAS Institute, Inc., Cary, NC, USA) [44].

## 3. Results

### 3.1. Population

**Figure 1:**
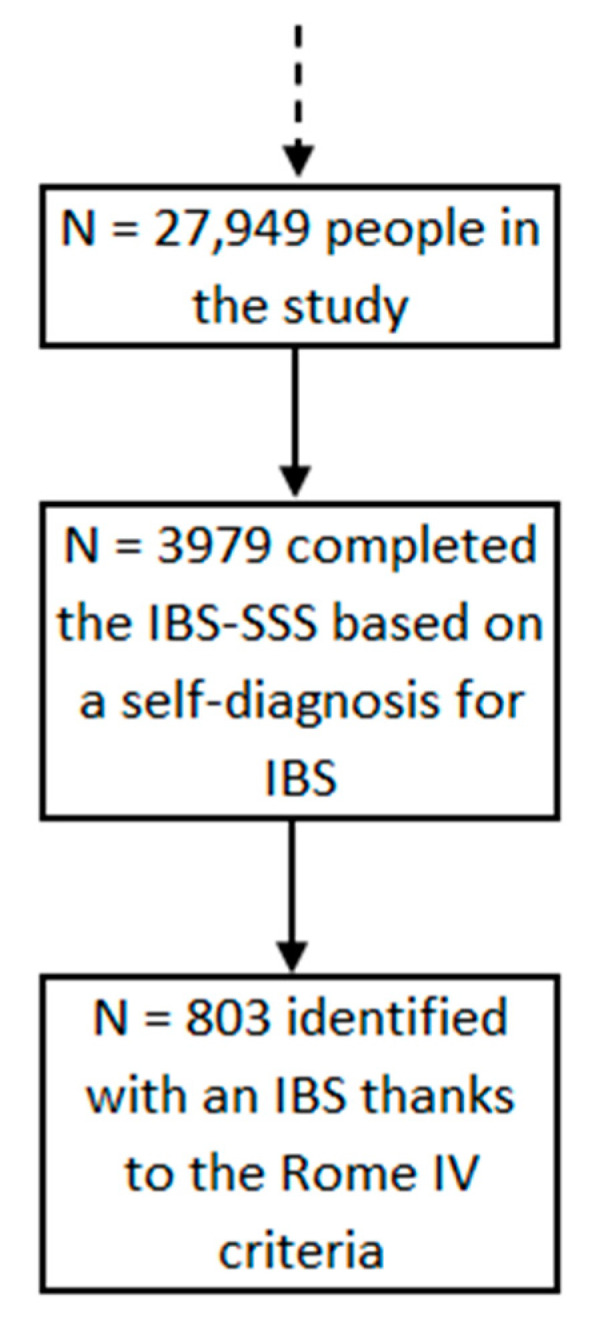
Flowchart of the IBS-SSS study, NutriNet-Santé cohort, France, 2009–2019 (N = 27,949).

The final sample included 27,949 people (Figure 1). Participants were mostly women (75.4%) with a mean age of 47.8 ± 14.1 years. Overall description of the population and comparison between non-IBS participants and IBS subjects using the Rome IV questionnaire is shown in Table 1.

### 3.2. Dietary Patterns and Proportion of IBS Cases in the Study Population

Overall 1295 (4.6%) subjects reported an IBS based on Rome IV criteria, with a higher proportion in women compared to men (5.44% in women vs. 2.17% in men, *p*-value < 0.0001). They were also more likely to be current smoker, younger, single, with a lower income (all *p*-value < 0.0001) and to have a healthier diet than non-IBS participants (*p*-value = 0.003) (Table 1).

IBS subjects tended to have lower energy intakes than non-IBS ones (1859 ± 488 kcal vs. 1906 ± 496 kcal, *p*-value < 0.0001). Their average total consumption of FODMAPs was 18.4 (SD = 9.6) g/day vs. 19.5 (SD = 9.5) g/day in non-IBS participants (Δ = −1.1 g/day, i.e., −6.0%, *p*-value < 0.0001). Moreover, IBS participants tended to have significantly lower intakes in excess fructose (Δ = −0.46 g/day, i.e., −11.0%), lactose (Δ = −0.2 g/day, i.e., −2.0%), GOS (Δ = −0.03 g/day, i.e., −8.3%), fructans (Δ = −0.12 g/day, i.e., −5.7%) and polyols (Δ = −0.22 g/day, i.e., −12.9%) (Table 2).

### 3.3. Association between FODMAP Consumption and IBS

After adjusting for covariates (model 3), an increase in total FODMAP intakes was associated with a reduced proportion of IBS (Table 3). The result was similar using FODMAP consumption as continuous (aOR: 0.88, 95% CI: 0.82–0.95, *p*-value: 0.001) or categorical: consumers with a high load of FODMAPs (i.e., >16 g/day) were less likely to be IBS patients in comparison with people with a low amount of FODMAPs (i.e., <9 g/day) (aOR: 0.82, 95% CI: 0.67–1.01, *p* for trend: 0.009) (Table 3). Sensitivity analyses were performed using 20 multiple imputations by the Chained equations method to handle missing values for the following variables: IPAQ (11.2%), income level (3.0%), residence (2.0%), educational level (0.8%), marital status (0.2%), smoking status (0.2%), BMI (0.2%) [45,46]. The results were similar, with an association between FODMAP consumption and IBS (model 3 aOR: 0.900 95% CI: 0.827–0.980) (data not tabulated).

### 3.4. FODMAP Consumption and IBS Severity

Out of 27,949 participants for this study, 3979 answered the IBS-SSS, based on a self-diagnosis for IBS. Among them, 20.2% (N = 803) met the Rome IV criteria for IBS.

Participants with an auto-diagnosed IBS were similarly distributed among each FODMAP category (<9 g/day, 9–16 g/day, ≥16 g/day) with the others. Indeed, 12% consumed less than 9 g/day of total FODMAPs in comparison with 54% who ate more than 16 g/day of total FODMAPs. The average total consumption of FODMAPs was 18.69 ± 9.55 g/day for those with an auto-diagnosis of IBS in comparison with 19.58 ± 9.55 g/day for the others (data not tabulated).

55% of IBS participants had at least moderate or severe symptoms according to the IBS-SSS (Table 4).

Among people who ate less than 9 g per day of total FODMAPs (N = 97, 12% of participants), the mean IBS-SSS was 228 ± 103. People who ate 9–16 g per day of total FODMAPs had an average IBS-SSS of 199 ± 97 whereas among people who ate more than 16 g per day of total FODMAPs (N = 430, 54% of participants), the mean IBS-SSS was 197 ± 98 (data not tabulated, p-value within each class 0.06).

**Figure 2:**
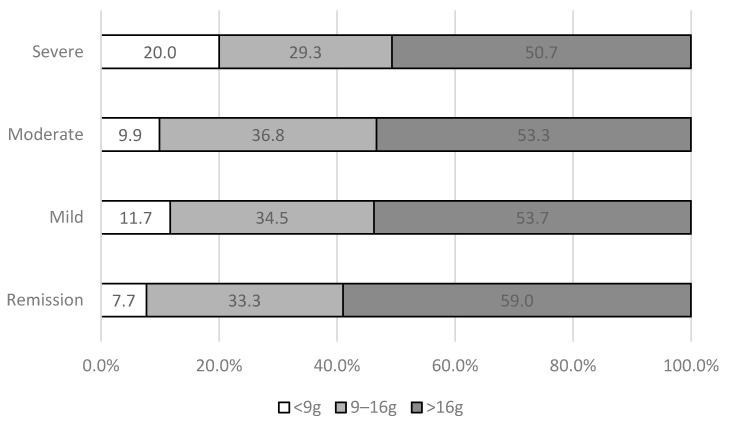
Proportion of IBS participants with different FODMAP intakes in different categories of severity according to the IBS-SSS, NutriNet-Santé cohort, France, 2009–2019 (N = 803).

Among severe cases of IBS participants, 20% ate less than 9 g of FODMAPs per day contrary to 51% of severe cases of IBS participants who ate more than 16 g of FODMAPs per day (Figure 2).

**Figure 3:**
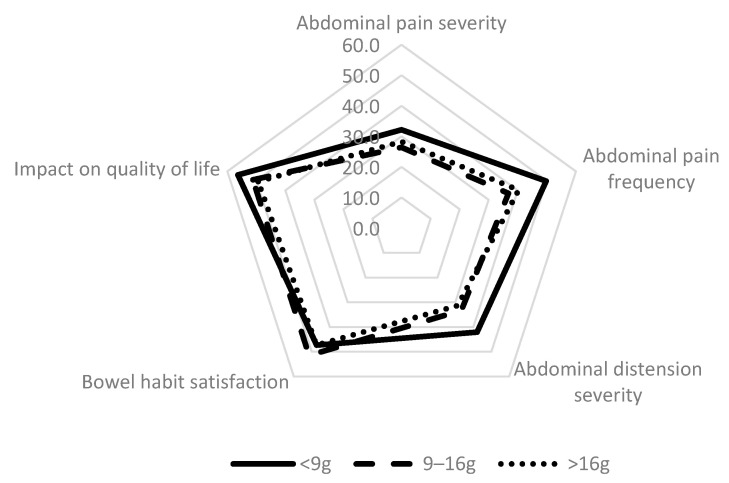
IBS-SSS individual items according to FODMAP intakes in IBS patients identified with the Rome IV criteria, NutriNet-Santé cohort, France, 2009–2019 (N = 803).

Focusing specifically on each IBS-SSS individual item, among people who were diagnosed with an IBS through the Rome IV criteria, those who consumed less than 9 g of total FODMAPs per day had more severe IBS symptoms in particular for abdominal pain and distension than the others (Figure 3).

## 4. Discussion

In this cross-sectional study performed in a large French cohort involving more than 27,000 people, we found that 4.6% of the participants had an IBS using the Rome IV criteria. Total FODMAP intake was around 19 g per day in both IBS and non IBS participants. However, IBS patients had a slightly lower intake of FODMAPs than non IBS participants and an increased severity of disease was associated with decreased intakes in FODMAPs.

The Rome IV proportion of 4.6% of IBS participants we found in this study compared with the 10.5% in our last study using the Rome III criteria [47] is consistent with a decrease in prevalence using the Rome IV criteria instead of the Rome III one [48,49].

To the best of our knowledge, this work is the first to assess FODMAP consumption in both IBS and non-IBS subjects in such a large population-based study. FODMAP intakes have already been studied in other countries in smaller studies. In Australia, an interventional study with a diet low in FODMAPs included 30 patients with IBS and 8 healthy controls and was associated with reduced functional gastrointestinal symptoms in patients with IBS [5]. In this study as well as in other studies, FODMAP intakes in both groups of IBS patients at baseline was similar at around 16 g per day [34,50,51]. These levels are of similar range but lower than the consumption we found in our study in both groups. Surprisingly, while we expected that the FODMAP content in the typical Australian diet would be higher than that of a French diet, it was not confirmed. The higher FODMAP consumption found in France in IBS subjects, around 19 g, compared with the 16.5 g per day of Australian and Swedish study is due to an increase in the excess fructose and polyol categories while lactose and GOS intakes are similar [5,50]. However, this total FODMAP intake of around 19 g in both groups is in accordance with our recent publication in more than 100,000 participants of the NutriNet cohort [32] and of 117 Swedish invidious representative of the general population [52].

In this study, although a small but significant difference in total FODMAP intake was found between IBS participants and controls, both intakes in FODMAPs are in the range of those known to be associated with symptoms in patients, i.e., over 9 g per day (32). These intakes and higher FODMAP intakes (over 16 g/day) are therefore susceptible to induce digestive symptoms in participants with a favoring profile for developing IBS such as a particular microbiota [53], visceral hypersensitivity [54] or altered intestinal permeability [55].

Interestingly, in our study, the mean daily intake in FODMAPs was slightly lower than that of non-IBS participants, and we found an inverse relationship between the level of FODMAP consumption and the prevalence of IBS; that is to say an increased intake in FODMAPs was associated with a lower prevalence of IBS. Reverse causality could partly explain these results, i.e., exposure to low loads of FODMAPs may be the consequence of IBS incidence. Indeed, we used a cross-sectional design (even though we tried to be as prospective as possible by choosing a dietary recall prior to the Rome IV questionnaire).

One hypothesis is that patients, aware of the low FODMAP diet (LFD) benefits on digestive symptoms, follow dietary advice to reduce their FODMAP load. This is confirmed by the fact that patients with a more severe disease according to global IBS-SSS or individual component of the score (pain, abdominal distension, impact on quality of life) were those with lower FODMAP consumption as if they were following a diet.

Given the nature of symptoms in IBS, directly linked in the patients’ mind to their diet [56], it may be difficult to disentangle causality in the relationship between FODMAPs and IBS in observational studies. However, it has been shown in the US that 60% of IBS patients described an association between consuming foods and GI symptoms and that they have frequent concerns about what food to avoid [57,58]. In people using the internet like our participants to an online study, information about disease is more frequently found on the internet and other media than in non-internet users, which could potentially lead to self-management [59,60]. A recent study in the US showed that half of the patients have tried an LFD before being seen by a gastroenterologist [58] and it was the same proportion in a recent study with patients from the French IBS association [61]. Furthermore, gastroenterologists themselves show a growing body of interest for diets, in particular the LFD: half of the providers now provide diet recommendations to over 75% of their IBS patients and they mostly recommend an LFD [58]. Indeed, most interventional studies showed a decrease in IBS severity after an LFD [5,24,50,51,62]. A prospective trial in the United States showed an improvement of quality of life in patients with diarrhea-predominant IBS with the very low carbohydrate diet in comparison with the standard American diet [63]. Conversely, a randomized, controlled, single-blinded trial in Sweden concluded that both an LFD and traditional IBS dietary advice improved IBS symptoms, but without any significant difference between the two dietary strategies [50].

Besides, though some have argued for a causal effect of FODMAP consumption on IBS symptoms, with small bowel luminal distension, colonic gas production varying with the type of FODMAP and influenced by gut microbiota [64], and visceral hypersensitivity [54], the association between FODMAP consumption and IBS is still controversial as seen elsewhere [22,50,65,66,67,68]. Another explanation could be that FODMAPs encompass lactose, and lactose intolerance is an analogous condition to IBS [69]. Moreover, FODMAP effects could be related to other food items such as fatty food or coffee that are not included in FODMAP definition [70,71].

This study has several strengths: First, a large number of participants were involved, allowing powerful analyses. Besides, for IBS definition, we used a validated questionnaire [10,72], as well as validated methods to assess FODMAP intakes [32]. The Rome IV questionnaire is also believed to select a more severe subpopulation from the IBS patients who were defined with the Rome III questionnaire [73,74,75]. Furthermore, socio-demographic characteristics of IBS patients in this study are consistent with the literature: IBS is more prevalent among young women and those with lower incomes [7,8,11]. This study also had a high specificity as the eligibility criteria—in particular regarding self-reported medical conditions and alert symptoms—was very restrictive, which probably led to the exclusion of healthy people. NutriNet-Santé study is a web-based study, thus enablingthe collection of data from various socio-demographic profiles, which allowed the use of multiple covariates to adjust for confounders. The results were adjusted for age, sex, energy, BMI, income per consumption unit, marital status, smoking status, overall diet quality, physical activity, residence and educational level, therefore minimizing some potentially confounding factors.

Several limitations of this study should be discussed. First, even though the work was designed to be as prospective as possible by choosing dietary records prior to the Rome IV questionnaire, it is still a cross-sectional study. Because of the observational design of the study, causal links cannot be asserted and the question of whether they were following a diet, and in particular an LFD was not directly asked to the participants. However, the hypothesis of reverse causality was reinforced by the analyses performed using the IBS-SSS. Indeed, since the LFD is increasingly known among IBS patients, they might have modified their dietary behavior before filling in the Rome IV questionnaire. The 9 g/day cut-off used in our study was chosen according to previous works of the Monash group [5,33] whereas the 16 g/day cut-off matches the usual daily amount of FODMAPs reached in several studies [5,34,50]. Further research from longitudinal studies could point out if FODMAP intakes tend to decrease after IBS onset and diagnosis. Another limitation relates to the voluntary profile of subjects, thus, subjects probably more interested in nutrition. They were therefore more likely to have a healthier diet compared to the general population, thus potentially leading to an underestimation of the associations that would be found in the general population. Consequently, caution is needed before generalization of the results. Another limitation is that the dietary records as well as the Rome IV questionnaire were administered by self-administered questionnaire. Nevertheless, this food collection system has been shown to be reliable [29,30,31]. Finally, even though potential confounding factors were taken into account, the study design does not enable to exclude the hypothesis of residual confounders.

## 5. Conclusions

In this large prospective population-based cohort, participants had a mean intake in FODMAPs of about 19 g per day. IBS patients had slightly lower intakes in FODMAPs than non-IBS participants. This may be due to reverse causality, as it was reinforced by the disease severity. Longitudinal studies are needed to better assess these findings, and to give clinicians new data on FODMAP intakes as a triggering factor for IBS symptoms.

## Figures and Tables

**Table 1:** Characteristics of participants according to IBS status, NutriNet-Santé cohort, France, 2009–2019 (N = 27,949).

Characteristics of Participants		All		IBS (Yes)		IBS (No)		*p*-Value
		N = 27,949		N = 1295 (4.63%)		N = 26,654 (95.37%)		
		n	%	n	%	n	%	
Age (years)	mean (SD)	47.8	(14.1)	43.4	(14.5)	48.0	(14.1)	<0.0001
Energy (kcal)	mean (SD)	1903	(495)	1859	(488)	1905	(496)	<0.0001
Sex	Men	6871	24.6	149	11.5	6722	25.2	<0.0001
	Women	21,078	75.4	1146	88.5	19,932	74.8	
Marital status	Single/divorced/widowed	7565	27.1	412	31.9	7153	26.9	<0.0001
	Married/cohabiting	20,327	72.7	881	68.1	19,446	73.1	
Educational level	No diploma or primary school	712	2.57	28	2.18	684	2.59	0.26
	Secondary school	8573	30.9	371	28.9	8202	31.0	
	Undergraduate	8372	30.2	407	31.7	7965	30.1	
	Graduate	10,059	36.3	479	37.3	9580	36.2	
Income per consumption unit (€/month)	Unwilling to answer	2034	7.50	78	6.27	1956	7.56	<0.0001
	<1110	3101	11.4	198	15.9	2903	11.2	
	1110–2330	17,932	66.2	821	65.9	17,111	66.2	
	>2330	4040	14.9	148	11.9	3892	15.0	
Residence	Rural area	6051	22.1	255	20.2	5796	22.2	0.10
	Urban area	21,350	77.9	1007	79.8	20,343	77.8	
Smoking status	Current smoker	3366	12.1	204	15.8	3162	11.9	0.0001
	Former	10,070	36.1	437	33.8	9633	36.2	
	Never	14,458	51.8	652	50.4	13,806	51.9	
BMI (kg/m^2^)	<18.5	1300	4.66	90	6.96	1210	4.55	<0.0001
	18.5–25	17,932	64.3	784	60.6	17,148	64.4	
	25–30	6355	22.8	276	21.3	6079	22.8	
	≥30	2319	8.31	144	11.1	2175	8.17	
IPAQ	Intense	8693	35.0	372	32.5	8321	35.1	0.16
	Moderate	10,705	43.1	508	44.3	10,197	43.0	
	Low	5433	21.9	266	23.2	5167	21.8	

BMI: Body Mass Index; IBS: Irritable Bowel Syndrome; IPAQ: International Physical Activity Questionnaire; SD: Standard Deviation; *p*-value: Chi-square tests or Student’s *t*-tests were used according to the qualitative or quantitative status of the characteristics; Missing data: Physical activity N = 3118 (11.2%); <5%: income level, residence, educational level, marital status, smoking status, BMI.

**Table 2:** FODMAP intake in the population under study according to the IBS, NutriNet-Santé cohort, France, 2009–2019 (N = 27,949).

	Global		IBS (Yes)		IBS (No)		*p*-Value
	N = 27,949		N = 1295 (4.6%)		N = 26,654 (95.4%)		
	Mean	SD	Mean	SD	Mean	SD	
Energy (kcal)	1903	495	1859	488	1906	496	<0.0001
Total FODMAPs (g/day)	19.4	9.5	18.4	9.6	19.5	9.5	<0.0001
Excess fructose	4.62	3.92	4.18	3.96	4.64	3.91	<0.0001
Lactose	10.3	7.5	10.1	7.3	10.3	7.5	<0.0001
GOS	0.39	0.37	0.36	0.38	0.39	0.37	<0.0001
Fructans	2.28	1.64	2.10	1.49	2.22	1.64	<0.0001
Polyols	1.92	2.05	1.71	2.00	1.93	2.06	<0.0001

IBS: Irritable bowel syndrome.

**Table 3:** Adjusted associations between FODMAP consumption and Irritable bowel syndrome, NutriNet-Santé cohort, France, 2009–2019 (N = 27,949).

	Continuous			Classes					
				≤9 g	9–16 g		>16 g		
				N = 2792	N = 8865		N = 16,292		
	OR	95% CI	*p*-Value		OR	95% CI	OR	95% CI	*p*-Trend
IBS									
Model 1	0.88	[0.83–0.94]	<0.0001	Ref	0.90	[0.75–1.09]	0.77	[0.64–0.92]	0.0005
Model 2	0.92	[0.86–0.98]	0.0066	Ref	0.93	[0.77–1.13]	0.82	[0.69–0.99]	0.0110
Model 3	0.88	[0.82–0.95]	0.0011	Ref	0.99	[0.80–1.22]	0.82	[0.67–1.01]	0.0086

*p*-values obtained with logistic regression using total FODMAPs as a continuous variable. *p*-values for trend obtained with logistic regression using classes as continuous variables. Model 1: Not adjusted; Model 2: Adjusted for age and sex; Model 3: Adjusted for model 2 + energy intake, BMI, income, marital status, smoking status, diet quality, physical activity, residence, and educational level.

**Table 4:** Proportion of IBS participants in different categories of severity according to the IBS-SSS, NutriNet-Santé cohort, France, 2009-2019 (N = 803).

IBS-SSS		n	%
Remission	0–75	78	9.7
Mild	75–175	281	35.0
Moderate	175–300	304	37.9
Severe	>300	140	17.4

## Data Availability

Data available upon request due to restrictions, e.g., privacy or ethical. The data presented in this study are available upon request from the corresponding author.
